# HIV-1 Capsid-Cyclophilin Interactions Determine Nuclear Import Pathway, Integration Targeting and Replication Efficiency

**DOI:** 10.1371/journal.ppat.1002439

**Published:** 2011-12-08

**Authors:** Torsten Schaller, Karen E. Ocwieja, Jane Rasaiyaah, Amanda J. Price, Troy L. Brady, Shoshannah L. Roth, Stéphane Hué, Adam J. Fletcher, KyeongEun Lee, Vineet N. KewalRamani, Mahdad Noursadeghi, Richard G. Jenner, Leo C. James, Frederic D. Bushman, Greg J. Towers

**Affiliations:** 1 University College London Medical Research Council Centre for Medical Molecular Virology, Division of Infection and Immunity, London, United Kingdom; 2 University of Pennsylvania School of Medicine, Department of Microbiology, Philadelphia, Pennsylvania, United States of America; 3 Medical Research Council Laboratory of Molecular Biology, Protein and Nucleic Acid Chemistry Division, Cambridge, United Kingdom; 4 HIV Drug Resistance Program, National Cancer Institute, Frederick, Maryland, United States of America; Vanderbilt University School of Medicine, United States of America

## Abstract

Lentiviruses such as HIV-1 traverse nuclear pore complexes (NPC) and infect terminally differentiated non-dividing cells, but how they do this is unclear. The cytoplasmic NPC protein Nup358/RanBP2 was identified as an HIV-1 co-factor in previous studies. Here we report that HIV-1 capsid (CA) binds directly to the cyclophilin domain of Nup358/RanBP2. Fusion of the Nup358/RanBP2 cyclophilin (Cyp) domain to the tripartite motif of TRIM5 created a novel inhibitor of HIV-1 replication, consistent with an interaction *in vivo*. In contrast to CypA binding to HIV-1 CA, Nup358 binding is insensitive to inhibition with cyclosporine, allowing contributions from CypA and Nup358 to be distinguished. Inhibition of CypA reduced dependence on Nup358 and the nuclear basket protein Nup153, suggesting that CypA regulates the choice of the nuclear import machinery that is engaged by the virus. HIV-1 cyclophilin-binding mutants CA G89V and P90A favored integration in genomic regions with a higher density of transcription units and associated features than wild type virus. Integration preference of wild type virus in the presence of cyclosporine was similarly altered to regions of higher transcription density. In contrast, HIV-1 CA alterations in another patch on the capsid surface that render the virus less sensitive to Nup358 or TRN-SR2 depletion (CA N74D, N57A) resulted in integration in genomic regions sparse in transcription units. Both groups of CA mutants are impaired in replication in HeLa cells and human monocyte derived macrophages. Our findings link HIV-1 engagement of cyclophilins with both integration targeting and replication efficiency and provide insight into the conservation of viral cyclophilin recruitment.

## Author Summary

During infection HIV-1 enters the nucleus by crossing the nuclear membrane and incorporating itself into the host DNA by a process called integration. Here we show that the viral capsid protein gets tethered to a cyclophilin protein called Nup358, a component of the nuclear membrane gateways that allow transport between the cytoplasm and the nucleus. Altering the capsid protein so that it cannot use Nup358 prevents viral replication in macrophages, a natural target cell type for HIV-1. Intriguingly, these viral mutants are not less infectious in certain immortalised cell lines suggesting that in these cells nuclear entry is regulated differently. In this case similar to wild type virus, the mutant viruses integrate into host chromosomes but they integrate into different regions suggesting that the pathway into the nucleus dictates where the virus ends up in the host chromatin. We also show that another cyclophilin, the cytoplasmic protein cyclophilin A, influences the engagement of Nup358 as well as other proteins involved in HIV-1 nuclear entry. We hypothesise that HIV-1 has evolved to use cyclophilins so that it can access a particular pathway into the nucleus because alternative pathways lead to defects in integration targeting and viral replication in human macrophages.

## Introduction

The ability to infect terminally differentiated cells of the monocyte-macrophage lineage is a conserved property of lentiviruses, including HIV-1 [1]. This process requires pre-integration complexes (PICs) to traverse the nuclear pore, though the molecular mechanism remains unclear. The HIV-1 proteins matrix, Vpr and integrase, as well as a DNA triplex at the central polypurine tract, have been proposed to contribute, but contrary evidence has been presented for each [2]–[5]. Gammaretroviruses such as murine leukemia virus (MLV) are dependent on cell division for infectivity and infect non-dividing cells inefficiently [6]. Characterization of HIV-1/MLV chimeric viruses has suggested a role for the HIV-1 capsid (CA) in nuclear entry [7]. Furthermore, certain HIV-1 CA mutants are selectively defective in arrested cells but not in actively dividing cells again implicating a role for CA in HIV-1 nuclear entry [8]–[10].

The nuclear pore complex (NPC), through which HIV replication intermediates must pass, consists of multiple copies of at least 30 different nuclear pore proteins (Nups). Nup358 is a large 358 kDa protein that constitutes the cytoplasmic filaments and has a C-terminal cyclophilin (Cyp) domain. It was first named Nup358 [11] but has also been called RanBP2 [12]. We use its original name Nup358 throughout this study. Several roles have been proposed for Nup358 involving cell cycle control, nuclear export, and transportin/importin dependent nuclear import (reviewed in [13]). In addition, Nup358 is a co-factor for HIV-1 replication, supporting nuclear entry of viral PICs and influencing target site preference for integration [14]–[17]. It has been unknown how the virus engages Nup358 and influences PIC traffic across the nuclear pore.

Here we demonstrate that HIV-1 CA binds directly to the Nup358 Cyp domain (Nup358Cyp) with an affinity within three fold of its binding of the monomeric cytoplasmic cyclophilin, CypA, which is known to be important during HIV-1 infection. We also demonstrate that CypA is important for directing HIV-1 into a nuclear entry pathway involving Nup358 and subsequent engagement of the nuclear basket protein Nup153, ensuring integration into preferred genomic loci. We report that altering CA interactions with Nup358 or CypA results in alterations in integration targeting preference, and reduced replication in macrophages. Our study provides the first evidence for direct interaction between HIV-1 CA and the NPC and suggests possible models for links between nuclear import, integration site selection and effective replication in primary human cells.

## Results

### Lentiviral Capsid Protein Determines Engagement of Nup358/RanBP2

**Figure 1:**
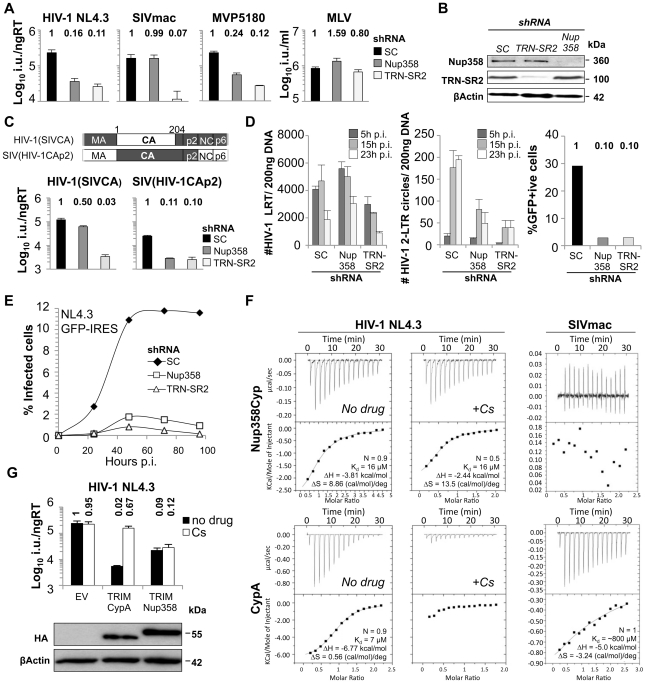
Binding of CA^NTD^ to Nup358 and effects on HIV-1 infectivity. **A**, VSV-G pseudotyped GFP encoding vectors derived from HIV-1 NL4.3 or MVP5180, SIVmac, or MLV were titered on HeLa cells expressing scrambled control (SC) or Nup358 or TRN-SR2 specific shRNA (mean and SD, n = 3). Relative changes from titer on control cells are shown above the bars. **B**, Western blots detecting Nup358, TRN-SR2 or β-Actin as loading control. **C**, Schematic representation of HIV-1/SIVmac chimeras and titration of HIV-1 GFP vector bearing an SIVmac CA (HIV-1 SIVCA) or SIVmac bearing HIV-1 CA-p2 (SIV(HIV-1CAp2)) measured on SC, Nup358, or TRN-SR2 depleted HeLa cells (mean and SD, n = 3). **D**, Measurement of HIV-1 late reverse transcription product (LRT) or 2-LTR circles at indicated time points post infection (p.i.) in HeLa control cells (SC) or cells depleted for Nup358 or TRN-SR2 (mean and SD, n = 3). A parallel sample was used to determine the number of infected cells by flow cytometry 48 h post infection. **E**, HeLa control cells (SC) or cells depleted for Nup358 or TRN-SR2 were transduced with an MLV vector expressing human CD4 and the neomycin resistance gene and drug selected cell population were infected with NL4.3GFP-IRES. Percentage of infected cells was enumerated at indicated time points by measuring GFP expression using flow cytometry. **F**, Isothermal titration calorimetry of cyclophilin A (CypA) or Nup358Cyp against the CA^NTD^ domains of NL4.3 HIV-1 or SIVmac in the presence or absence of 10 µM cyclosporine (Cs). **G**, HIV-1 GFP vector titer on CRFK cells expressing empty vector (EV), owl monkey TRIM5 RBCC fused to human CypA (TRIMCypA) or human Nup358Cyp (TRIMNup358) in the presence or absence of 5 µM Cs (mean and SD, n = 3). Protein levels measured by western blot detecting the HA tag with β-Actin as a loading control.

Several studies have shown that depletion of Nup358 reduces HIV-1 infectivity. We sought to define the HIV-1 determinant that confers its sensitivity to Nup358 depletion by studying infections with VSV-G pseudotyped viral vectors encoding GFP. Stable Nup358 depletion by transduction of HeLa cells with MLV or HIV-1 based shRNA expression vectors reduced HIV-1 GFP vector infectivity by 6- to 8-fold confirming Nup358's role as an HIV-1 cofactor [14], [17] (Figure 1A, B and Figure S1). We validated effective shRNA targeting by western blotting, using a Nup358 specific antibody (Figure 1B), as well as by co-transfecting the shRNA expression vector and a plasmid encoding GFP-tagged Nup358 into 293T cells (Figure S2). Studies on the role of Nup358 in HIV-1 replication have used M-group HIV-1 isolates [14]–[17]. In order to confirm the importance of Nup358 as a cofactor for other HIV-1 isolates we also tested the O-group HIV-1 virus MVP5180 [18], [19] as a distantly related HIV-1 and found that this too was sensitive to Nup358 depletion, suggesting that Nup358 use is a conserved feature of HIV-1 biology (Figure 1A and Figure S1C). We next tested whether the even more distantly related simian immunodeficiency virus from macaques (SIVmac) was sensitive to Nup358 depletion. In contrast to HIV-1, infectivity of SIVmac, was not reduced by Nup358 RNAi, suggesting species-specificity of Nup358 use (Figure 1A and Figure S1C). We next sought to identify the viral determinant for Nup358 RNAi sensitivity. Given that the HIV-1 capsid protein (CA) has been implicated in HIV-1 nuclear import [7], [8], [10], we tested whether the different sensitivities to Nup358 depletion between HIV-1 and SIVmac could be accounted by their different CA proteins. We exchanged CA coding regions between HIV-1 and SIVmac and analyzed infectivities of chimeric viruses on Nup358 depleted cells. Replacement of SIVmac CA with CA from HIV-1 [20] rendered the chimeric SIVmac sensitive to Nup358 depletion, while replacement of HIV-1 CA with SIVmac CA [21] rendered HIV-1 largely insensitive to Nup358 depletion (Figure 1C). For comparison, we examined the sensitivity of these viruses to transportin 3 (TRN-SR2) depletion, and confirmed that TRN-SR2 specific shRNA reduced infectivity of both HIV-1 (∼8 to 10-fold) and SIVmac (∼20-fold) (Figure 1A and Figure S1). MLV GFP vector infectivity was not affected by depletion of these proteins as reported previously, consistent with MLV's inability to traverse the nuclear pore and infect non-dividing cells (Figure 1A and Figure S1).

If Nup358 and TRN-SR2 facilitate nuclear entry of wild type HIV-1, then their depletion should inhibit HIV-1 infection at the level of nuclear import. We confirmed that 2-LTR circle products of HIV-1 were modestly reduced in abundance in the Nup358 or TRN-SR2 depleted cells, whereas late reverse transcript production was unaffected (Figure 1D) [22]. However, we observed that the ten-fold reduction in infectivity was greater than the two to four-fold reduction in 2-LTR circles, possibly explained by an integration defect increasing the amount of 2-LTR circles. To measure integration we infected Nup358 or TRN-SR2 depleted cells with HIV-1 GFP vector, grew the cells for 2 weeks and measured the number of integrated proviruses by Taqman qPCR. We observed that the reduction of integrated proviruses in Nup358 depleted cells (5-fold) was similar to the reduction of 2-LTR circles (4-fold) (Figure 1D and Figure S3). In contrast, the reduction of proviruses in TRN-SR2 depleted cells was significantly greater (50-fold), than the reduction in 2-LTR circles (2 to 3-fold). This observations may suggest that Nup358 depletion blocks HIV-1 at a step prior to nuclear import but after reverse transcription, whereas TRN-SR2 depletion imposes two blocks, one at the stage of nuclear import (reduction of 2-LTR circles) and a second at integration. However, we suggest caution in interpretation of 2-LTR circle assay as a measure of nuclear entry given that 2-LTR circles are non productive for infection and their formation may have different co-factor requirements. Importantly, replication of wild type NL4.3GFP-IRES was also impaired in Nup358 or TRN-SR2 depleted HeLa cells expressing CD4 (Figure 1E). Equivalent CD4 expression in these cells was confirmed by flow cytometry using fluorescent CD4 specific antibody (Figure S4). These data suggest that Nup358 and TRN-SR2 contribute to optimal viral nuclear entry, integration and eventually replication.

### Nup358 Interacts with HIV-1 CA via Its Cyclophilin Domain

Nup358 contains a cyclophilin domain (Nup358Cyp) at its extreme carboxyl-terminus. The HIV-1 N-terminal CA domain (CA^NTD^) resides on the surface of the virion core and recruits CypA to viral cores [23], [24]. The CA-dependent sensitivity of HIV-1 to Nup358 depletion led us to hypothesize that HIV-1 CA^NTD^ might also interact with Nup358Cyp in a similar manner to its interaction with CypA. To test this, we purified recombinant CypA and Nup358Cyp and measured binding to recombinant CA^NTD^, using isothermal titration calorimetry (ITC) [25]. We found that the HIV-1 CA^NTD^ bound Nup358Cyp with a Kd of 16 µM, in a similar range to its Kd of 7 µM for CypA (Figure 1F) [25]. Surprisingly, the CypA inhibitor cyclosporine (Cs) did not prevent Nup358Cyp binding to HIV-1 CA^NTD^ whereas it did inhibit CypA binding. Whilst capsid interaction with both Nup358Cyp and CypA was entropically favourable, interaction with Nup358Cyp was more strongly entropically favourable than CypA. This does not markedly alter the affinity with respect to CypA as CA^NTD^ interaction with CypA is more enthalpically favourable than with Nup358Cyp. The different thermodynamic signatures between CypA and Nup358Cyp suggest that the two proteins do not form identical interactions. The entropic component of any interaction is the sum of changes in protein and solvent dynamics. Given that the ligand, CA^NTD^, is the same in each experiment whilst Nup358Cyp and CypA comprise a single globular fold it is likely that the entropically favourable nature of both interactions is a consequence of releasing ordered water molecules upon complex formation. The larger entropic change associated with Nup358Cyp interaction may indicate a greater release of ordered water upon complexation, suggesting that the interface is larger than in CypA:capsid.

SIVmac CA^NTD^ did not bind Nup358Cyp (Figure 1F), and bound CypA with a very low affinity (∼800 µM) (Figure 1F), which becomes important below. The inability of Nup358Cyp to bind to SIVmac CA^NTD^ correlates with the insensitivity of SIVmac to Nup358 depletion in HeLa cells (Figure 1A).

To probe Nup358Cyp binding further we designed an HIV-1 inhibitor based on the simian restriction factor TRIMCyp. Owl monkey TRIMCyp blocks HIV-1 by binding incoming capsids via its CypA domain [26]. We replaced the TRIMCyp cyclophilin domain with human CypA or Nup358Cyp to make TRIMCypA and TRIMNup358. We found that both TRIMNup358 and TRIMCypA blocked HIV-1 infectivity (Figure 1G) whereas SIVmac infection was not restricted, as expected from the lack of binding (Figure S5). Importantly, Cs treatment only rescued infectivity from TRIMCypA but not from TRIMNup358, corroborating Cs sensitivity measured by ITC (Figure 1F). We confirmed similar expression levels of chimeric proteins by western blot (Figure 1G). These data are consistent with HIV-1, but not SIVmac, CA^NTD^ efficiently binding Nup358Cyp in the context of TRIMNup358 in the cytoplasm of infected cells. This suggests that HIV-1 PICs containing CA or possibly entire capsid cores can interact directly with a component of the NPC, providing insight into how HIV-1 contacts the nuclear pore during the process of nuclear entry.

### Residue 61 of the Nup358 Cyclophilin Domain Is Positively Selected

**Figure 2:**
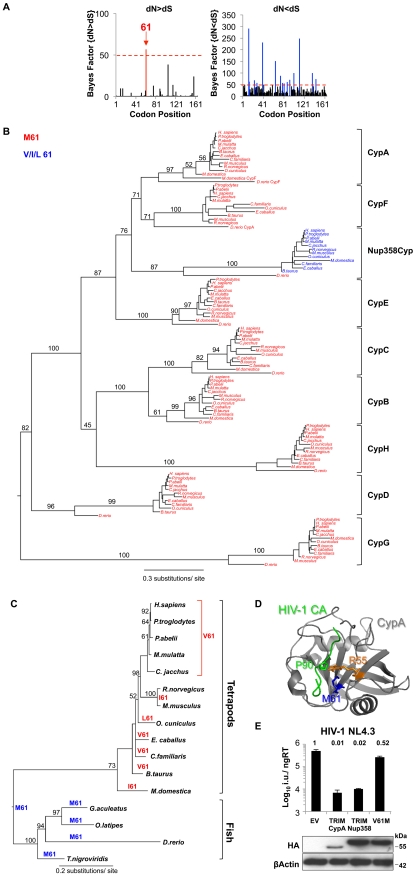
Nup358Cyp evolved under positive selection pressure. **A**, Codon positions under positive (dN>dS) and negative (dN<dS) selection along the Nup358Cyp gene sequence, as indicated by the Bayesian probability that a particular site is under selective pressure. A Bayes factor >50 is considered strong evidence for the favored model. **B**, Maximum likelihood phylogenetic tree of cyclophilins A, B, C, D, E, F, G, H and Nup358Cyp from Rhesus macaque (*M. mulatta*), Human (*H. sapiens*), Chimpanzee (*P. troglodytes*), Dog (*C. familiaris*), Cow (*B. taurus*), Horse (*E. caballus*), Marmoset (*C. jacchus*), Mouse (*M. musculus*), Rat (*R. norvegicus*), Opossum (*M. domestica*), Zebrafish (*D. rerio*), Orangutan (*P. abelii*) and Rabbit (*O. cuniculus*). Branch lengths and bootstrap supports are shown. **C**, Maximum likelihood phylogenetic tree demonstrating the simplest model of evolution of residue 61 in Nup358Cyp sequences. **D**, Structure of CypA bound to CA^NTD^ with CypA key residues R55 and M61 as well as HIV-1 CA P90 highlighted. (PDB:1FGL) **E**, NL4.3 HIV-1 GFP vector titer on CRFK cells expressing empty vector (EV), TRIMCypA, wild type TRIMNup358, or TRIMNup358 mutant V61M. Protein levels measured by western blot detecting the HA tag with β-Actin as loading control are shown. The data are representative of three independent experiments (mean and SD, n = 3).

Host proteins that interact with pathogens are often under positive selective pressure [27]. A higher rate of non-synonymous nucleotide substitutions (dN) than synonymous substitutions (dS) at a particular codon in an interspecies comparison provides evidence for such positive selection. We aligned Nup358Cyp DNA sequences from 12 different species (Figure S6) and performed an analysis of codon-specific selective pressures using the program Random Effect Likelihood (REL) implemented on the online version of the HyPhy package [28]. Despite overall strong negative selection across the Cyp domain, we found Nup358Cyp codon 61 to be positively selected at a statistically significant level (Bayes factor >50; Figure 2A). Indeed, residue 61 is extremely conserved as methionine across the whole vertebrate Cyp family except in Nup358Cyp. (Figure 2B, C). In the case of Nup358Cyp lower vertebrates encode the ancestral methionine, whereas higher vertebrates encode valine, leucine or isoleucine at this position. Fixation of the positively selected site appears to have occurred after the divergence of fish and tetrapods, since Nup358Cyp sequences from fish (e.g. *Danio rerio*) retain methionine at position 61 (Figure 2B, C). This observation suggests that Nup358 has been under selective pressure to evolve and that this has led to variation in the sequence at this position. The cyclophilin domain of Nup358 has been proposed to possess prolyl *cis*-*trans* isomerase activity, similarly to CypA [29], [30]. Assuming that the Nup358Cyp active site is homologous to that of CypA then according to the CypA structure (PDB:1FGL) residue 61 is located directly at the bottom of the active site suggesting that it might impact on substrate specificity (Figure 2D). To examine this further we made a TRIMNup358 mutant in which the valine at Nup358Cyp position 61 was changed to the ancestral residue methionine. TRIMNup358 V61M was no longer able to restrict HIV-1, suggesting that binding to HIV-1 CA is influenced by this residue (Figure 2E). Our results suggest that during evolution selective pressure, possibly from ancient pathogenic viruses, has driven the change of Nup358Cyp position 61, altering substrate specificity in higher vertebrates. In turn HIV-1 is adapted to use this modified protein for nuclear entry in humans.

### Substitutions in HIV-1 CA^NTD^ Alter Engagement of Nup358/TRN-SR2

**Figure 3:**
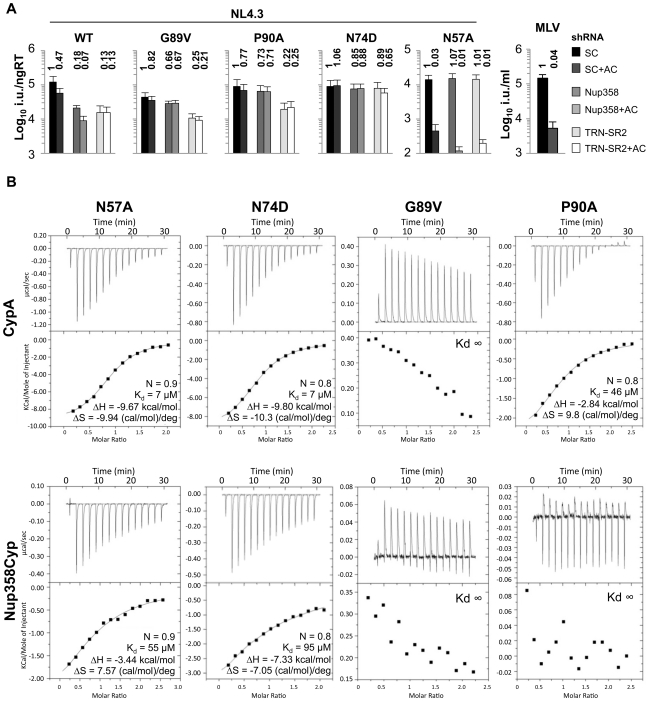
Effect of CA mutants on Nup358/TRN-SR2 RNAi sensitivity and Nup358Cyp binding. **A**, Titers of VSV-G pseudotyped NL4.3 HIV-1 GFP vectors bearing wild type or mutant CA on HeLa control cells (SC), or Nup358 or TRN-SR2 depleted cells in the presence or absence of 2 µg/ml aphidicolin (AC). MLV GFP vector was used as a control for aphidicolin. Relative changes from titer on control cells are shown above the bars. The data are representative of two independent experiments each using three different virus doses. **B**, ITC of human CypA and Nup358-Cyp against HIV-1 CA^NTD^ mutants. K_d_ values as well as stoichiometries (N), enthalpies (ΔH) and entropies (ΔS) are shown.

If binding of HIV-1 CA to Nup358 is important for HIV-1 infectivity, then amino acid substitutions in CA that affect interaction with Nup358 should influence infectivity. Indeed, we found that whilst wild type HIV-1 infectivity is sensitive to both Nup358 as well as TRN-SR2 depletion, certain HIV-1 CA mutants were not, suggesting an inability to utilize these cofactors. We infected HeLa cells stably expressing Nup358 or TRN-SR2 shRNA with GFP-encoding VSV-G pseudotyped HIV-1 vectors bearing wild type or mutant CA. We found that the cyclophilin-binding mutants G89V and P90A were insensitive to Nup358 depletion but remained sensitive to TRN-SR2 depletion (Figure 3A). We hypothesized that an inability to bind Nup358Cyp or CypA might underlie infectivity defects of other HIV-1 CA mutants. The HIV-1 CA mutant N57A is more severely defective in arrested cells than dividing cells (Figure 3A) [10], suggesting that this residue may have a role in nuclear entry. Indeed, ITC demonstrates that N57A is impaired in binding Nup358Cyp (Kd 55 µM) but not CypA (Kd 7 µM) (Figure 3B). As, N57A is less sensitive to both Nup358 and TRN-SR2 depletion (Figure 3A), we hypothesize that its infectivity defect is caused by an inability to engage these proteins. We found that N57A was still restricted by TRIMNup358 (Figure S5), suggesting that increased avidity through Nup358Cyp dimerization in the context of TRIMNup358 may overcome the reduced affinity to monomeric Nup358Cyp. Importantly, N57A's insensitivity to Nup358 depletion suggests that it does not engage Nup358 during nuclear entry.

**Figure 4:**
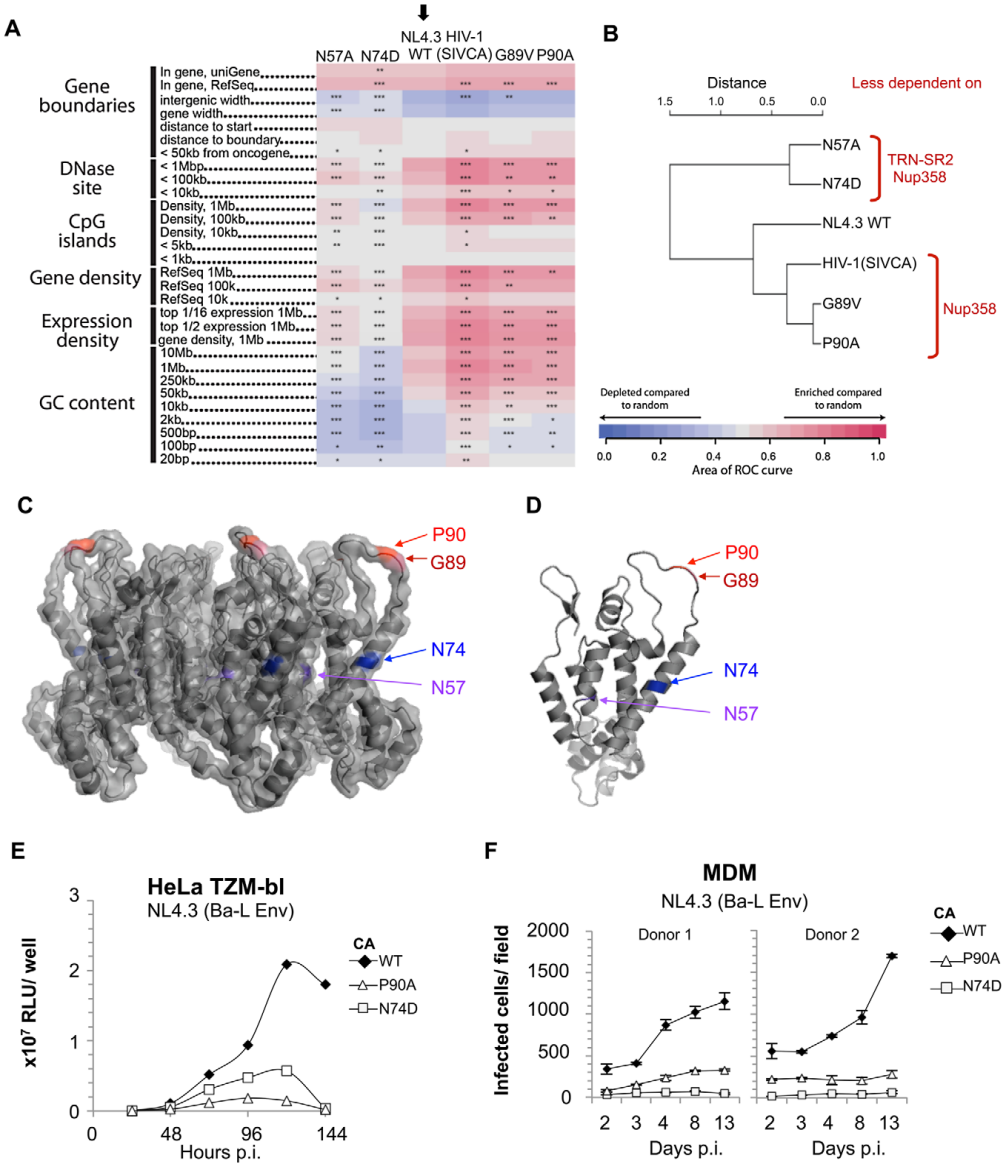
Residue changes in HIV-1 CA mutants alter integration site targeting. **A**, Effects of CA mutants on integration frequency near multiple chromosomal features. The rows show genomic features, and the columns indicate integration site data sets. The color code indicates ROC areas [34]. The arrow denotes the wild type control set used for pairwise statistical comparisons to the other data sets. P values summarizing the significance of departures from the control are represented by asterisks (*P<0.05; **P<0.01; ***P<0.001). Several different intervals are compared. "Expression density" summarizes the density of genes in the indicated length intervals that are expressed in the upper half ("top 1/2") or upper sixteenth ("top 1/6") of all genes on Affymetrix HU95A chip data for HeLa cells (accessions GSM23372, GSM23373, GSM23377, and GSM23378). For a more detailed guide see [16]. **B**, ROC area values were used to generate pairwise Euclidean distances, which were then analyzed by hierarchical clustering generating the presented dendogram. The locations of changed residues in mutant viruses studied on **C**, the CA hexamer (PDB:3GV2) and **D**, the CA monomer (PDB:3GV2). **E**, Time course of replication competent NL4.3 (Ba-L Env) bearing wild type, P90A or N74D CA in HeLa TZM-bl cells. Luciferase expression was measured by counting RLU at indicated times after virus inoculation. The data are representative of two independent experiments. **F**, Replication assay of NL4.3 (Ba-L Env) bearing wild type or CA mutants P90A or N74D in human MDM. Cells were stained for Gag p24 at specific time points after infection and infected colonies counted. Standard errors of the mean of three fields are shown.

Finally, we studied the HIV-1 CA mutant N74D, which is reported to be less sensitive to Nup358 or TRN-SR2 depletion (Figure 3A) [17], [31]. Like N57A, N74D bound monomeric Nup358Cyp in ITC experiments with significantly lower affinity than wild type (Kd 95 µM) (Figure 3B) and like N57A, N74D was also restricted by TRIMNup358 (Figure S5A). As for N57A, it seems contradictory that HIV-1 CA mutants that are less sensitive to Nup358 depletion are restricted by TRIMNup358. We assume that binding characteristics of TRIMNup358, and Nup358 itself, to CA are different particularly given that TRIM5 is reported to form cytoplasmic dimers [32] and higher-order multimers [33], Thus a forced dimerization of Nup358Cyp by fusing it to TRIM5α, could increase binding of Nup358Cyp to CA by increasing avidity, thereby allowing restriction. In addition, it is possible that the decreased binding of N57A as well as N74D to TRIMNup358 is disguised by an increased sensitivity to restriction by this TRIM5 chimera. HIV-1 CA N57 is located at the base of helix 3 and N74 in helix 4 (Figure 4C, D), suggesting that amino acid residues outside the Cyp-binding loop can impact on Nup358Cyp binding. We propose that Nup358 and TRN-SR2 define an import pathway used by wild type HIV-1 and that CA amino acid substitutions direct the virus to use Nup358 independent (G89V, or P90A), or Nup358/TRN-SR2 independent (N74D, or N57A) import pathways.

To test whether HIV-1 dependence on Nup358 is increased in non-dividing cells, we arrested HeLa cells with aphidicolin and measured infectivity of the CA mutant viruses. We found that only N57A and MLV infectivities were inhibited by aphidicolin treatment (Figure 3A and Table S1) whereas mutants G89V, P90A and N74D were not affected. This suggests that G89V, P90A and N74D use Nup358/TRN-SR2 independent routes into the nucleus even in the absence of cell division. This hypothesis is supported by the observation that neither the wild type virus nor these mutants become additionally sensitive to Nup358 or TRN-SR2 RNAi in aphidicolin-arrested cells (Figure 3A and Table S1). On the other hand N57A is slightly increased in its sensitivity to aphidicolin particularly after Nup358 depletion. We conclude that these co-factors are required for HIV-1 infection of dividing and non-dividing cells.

### Nup358/TRN-SR2 Independent HIV-1 CA Mutants Integrate with Different Integration Site Preferences

HIV integration is favored in chromosomal regions rich in genes and associated features such as CpG islands, DNAaseI hypersensitive sites, and high G/C content. We have shown that Nup358 or TRN-SR2 depletion reduces HIV-1 integration frequency near these features [16]. To test this for the CA mutants studied above, we sequenced 19,546 unique integration sites from HIV-1 and its mutants by 454/Roche pyrosequencing and compared their chromosomal distributions as described [16], [34]–[37].

Although the HIV-1 CA mutants retained the preference for integration within transcription units, their integration site distributions diverged from wild type HIV-1. The patterns clustered into two groups that map to two distinct areas on the CA surface (Figure 4). HIV-1 CA mutants N57A and N74D integrated into regions of chromatin associated with a significantly lower density of transcription units and associated features. For wild type HIV-1 this density was 15 transcription units/MB, whereas for CA mutants N57A or N74D the density was reduced to what is expected for random integration (7–9 transcription units/MB) (Figure 4A and Figure S7). In contrast, the two Cyp-binding mutants, G89V and P90A, exhibited an opposite phenotype, with favored integration into regions of increased density of transcription units (∼20 transcription units/MB). The chimeric HIV-1 containing SIVmac CA, showed a further increased preference for regions dense in transcription units (25 transcription units/MB) (Figure 4A and Figure S7A). These latter three viruses similarly showed increased frequency of integration in areas rich in active genes, CpG islands, DNase sites, and high in GC content, features correlating with high gene density. Hierarchical clustering of the CA mutants based on these data separated the viruses into two groups: N57A and N74D, and the Cyp-binding mutants G89V, P90A and chimeric HIV-1(SIVCA) (Figure 4B). Wild type HIV-1, which has an intermediate targeting phenotype, was an outlier within this second group. Thus amino acid substitutions in CA can alter integration targeting preference, resulting in either of two phenotypes. Because G89V and P90A influence targeting in the same direction, we infer that disruption of normal CypA interactions, and possibly Nup358 interactions, result in increased frequency of integration in regions with high densities of transcription units. The N74D and N57A substitutions are less sensitive to depletion of both Nup358 and TRN-SR2, and N74D gains sensitivity to depletion of other nuclear pore proteins [17]. We thus infer that this pathway leads to favored integration in regions with lower densities of transcription units.

We were surprised that HIV-1 CA mutants P90A and N74D, which are less sensitive to depletion of TRN-SR2 and/or Nup358 (Figure 3A), and have different integration site preferences in unmodified cells (Figure 4), were as infectious as wild type virus in single round assays. This is true when the virus is pseudotyped with the VSV-G envelope (Figure 3A) or the natural HIV-1 gp160 envelope (Figure S9). To test whether these CA substitutions affect HIV-1 replication we compared replication of wild type HIV-1 NL4.3 (Ba-L Env) with CA mutants P90A and N74D in spreading infection in HeLa TZM-bl cells [38]. Interestingly, we found that replication of both HIV-1 CA mutants was impaired in these cells compared to wild type virus, suggesting that cofactors used for nuclear entry and/or integration site selection are important for optimal replication (Figure 4E). We also found that HIV-1 NL4.3 (Ba-L Env) bearing CA alterations N74D or P90A replicated poorly in primary human MDM from four independent donors, whereas wild type virus replicated efficiently (Figure 4F and Figure S7B). These data demonstrate that HIV-1 CA mutants P90A and N74D do not support optimal replication. One possible explanation is that this is due to differences in their integration site targeting as compared to the wild type virus, though other models are possible. Whether the defect in replication is due to a defect in viral gene expression remains unclear. However, it is clear that the mutant viruses that are unable to effectively utilize Nup358 or TRN-SR2 display a replication defect in a cell line and in primary human macrophages.

### CypA-CA Interactions Dictate the Use of a Nup358/Nup153 Dependent Nuclear Entry Pathway

**Figure 5:**
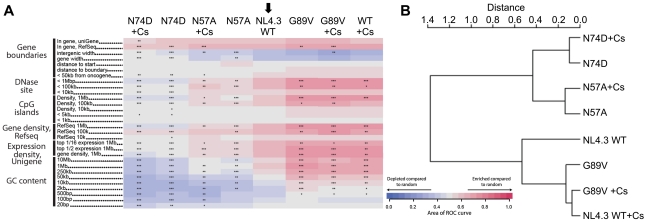
CypA inhibition alters HIV-1 integration site targeting. **A**, Effects of CypA inhibition by cyclosporine (Cs) on integration frequency near multiple chromosomal features for wild type NL4.3 HIV-1 GFP vector and CA mutants. The arrow denotes the wild type control set used for pairwise statistical comparisons to the other data sets. The heat map analysis was performed as in Figure 4A. **B**, ROC area values were used to generate pairwise Euclidean distances, which were then analyzed by hierarchical clustering generating the presented dendogram.

The observation that HIV-1 CA mutants P90A and G89V, as well as chimeric HIV-1(SIVCA) integrate into genome regions with higher densities of transcription units and associated features raised the possibility that integration targeting might be influenced by CypA binding to CA. Since cyclosporine (Cs) selectively inhibits CypA but not Nup358Cyp binding (Figure 1F, G), we investigated whether Cs could retarget integration by HIV-1. In fact, Cs treatment retargeted viral integration preferences in a way that phenocopied the CA G89V/P90A substitutions shifting integration preferences into regions of higher gene density (Figure 5A, B). Thus preventing CypA-CA interactions with Cs has the same effect on integration targeting as amino acid substitutions in HIV-1 CA that block CypA binding, supporting the idea that integration targeting is truly affected by cyclophilin-CA interactions.

**Figure 6:**
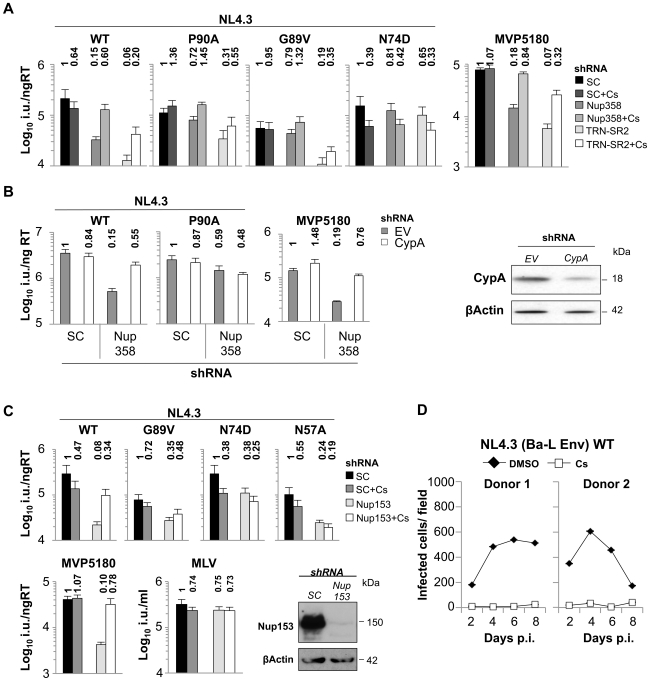
CypA inhibition forces HIV-1 into a Nup358/Nup153 independent nuclear entry pathway. **A**, Titers of VSV-G pseudotyped NL4.3 HIV-1 GFP vectors bearing wild type or mutant CA on HeLa control cells (SC), or Nup358 or TRN-SR2 depleted cells in the presence or absence of 8 µM Cs (mean and SD, n = 3). Relative changes from titer on control cells are shown above the bars. **B**, HeLa cells expressing scrambled control (SC), or Nup358 specific shRNA were transiently transduced with either MLV empty vector control or vector expressing CypA specific shRNA and subsequently infected with wild type NL4.3 HIV-1 GFP vector or CA mutant P90A or O-group MVP5180 GFP vector. Infection was measured 48 hours later by FACS to enumerate infected cells. Fold changes to control cells are shown. A parallel western blot for experiments in B detecting CypA and β-Actin as loading control. **C**, HeLa control cells (SC) or transiently Nup153 depleted cells were infected with indicated vectors in the presence or absence of 8 µM Cs (Mean and SD, n = 3). Western blot detecting Nup153 and β-Actin as a loading control. **D**, MDM from 2 independent donors were infected with 400 pg RT replication competent HIV-1 NL4.3 (Ba-L Env) in the presence of 5 µM Cs or DMSO. Replication was measured over time by counting CA positive cells after staining with a CA specific antibody.

Reduction of Nup358 by RNAi led to integration into low gene density/activity regions [16] but preventing CypA binding by CA amino acid substitutions (G89V/P90A) or Cs treatment shifted virus integration preferences into high gene density/activity regions. This suggested to us that Nup358 and CypA have different, possibly opposing effects on HIV-1. Alternately, Cs treatment may somehow change the availability of Nup358 in the cell. To investigate this further we tested whether CypA inhibition in Nup358 depleted cells influences HIV-1 infectivity. Remarkably, Cs treatment specifically rescued HIV-1 infectivity reduced by Nup358 depletion to the level observed in control cells (Figure 6A and Figure S8). We note that the small inhibitory effect of Cs on HIV-1 infectivity is preserved and infectivity is rescued to the level of infectivity on control cells treated with Cs. Thus Cs inhibits HIV-1 GFP infectivity by 2–3 fold but concomitantly rescues infectivity from the effects of Nup358 depletion. Transient CypA depletion using shRNA expression had a similar effect as CypA inhibition with Cs, also rescuing infectivity reduced by Nup358 depletion (Figure 6B). As expected, the CypA insensitive mutants G89V or P90A did not respond significantly to Cs treatment or CypA depletion by RNAi respectively (Figure 6A, B). The infectivity of the HIV-1 CA mutant N74D was slightly reduced by Cs consistent with its reduced sensitivity to Nup358 depletion and supporting the notion that it is still able to recruit CypA as confirmed by ITC (Figure 3B, 6A and Figure S8). Cs also partially rescued HIV-1 infectivity in cells with strong TRN-SR2 depletion (Figure 6A and Figure S8), suggesting that TRN-SR2 participates in the Nup358 dependent import pathway into which the virus is directed by CypA.

We were also able to show that the distantly related HIV-1 O-group virus MVP5180 was also specifically rescued upon Cs treatment/CypA depletion in Nup358 or TRN-SR2 depleted cells but was unaffected in control cells (Figure 6A, B). This suggests that MVP5180 functionally interacts with CypA in a similar way to NL4.3 and this is concordant with the very similar co-crystal structures of M-group HIV-1 CA^NTD^ with CypA and O-group HIV-1 CA^NTD^ with CypA (PDB ID: 1M9D) [39]. Together these observations made using both NL4.3 and MVP5180 suggest that CypA acts upstream of Nup358 and that Nup358 is not required for HIV-1 infectivity in the absence of CypA activity. In other words we propose that CypA activity directs the virus to engage Nup358.

If CypA activity directs HIV-1 to interact with cytoplasmic Nup358 to traverse the NPC then reduced HIV-1 infectivity through depletion of nuclear pore proteins that act downstream of Nup358 should also be rescued by CypA inhibition. To test this we analyzed infectivity of HIV-1 NL4.3 and its CA mutants in HeLa cells depleted for Nup153 (Figure 6C). Nup153 is a NPC component in the nuclear basket and has been highlighted in genome wide siRNA screens as co-factor for HIV-1 [14], [40], [41]. We found that HIV-1 NL4.3 infectivity was strongly reduced in Nup153 depleted cells by ∼10-fold, whereas MLV infection was not affected (Figure 6C). However, the Cyp non-binding mutant HIV-1 CA G89V as well as mutants N74D and N57A, which are less dependent on Nup358/TRN-SR2 were only moderately affected (∼3-fold). When cells were treated with Cs during infection, infectivity reduced by Nup153 depletion was specifically rescued for wild type virus, whereas the HIV-1 CA mutants remained unaffected (Figure 6C). The O-group HIV-1 MVP5180 was affected by Nup153 depletion similarly to NL4.3 and CypA inhibition rescued its infectivity similarly to what we observed in Nup358 depleted cells. These observations support the notion that inhibition of CypA recruitment leads HIV-1 to use different cellular cofactors, and perhaps a different pathway, for nuclear entry.

**Figure 7:**
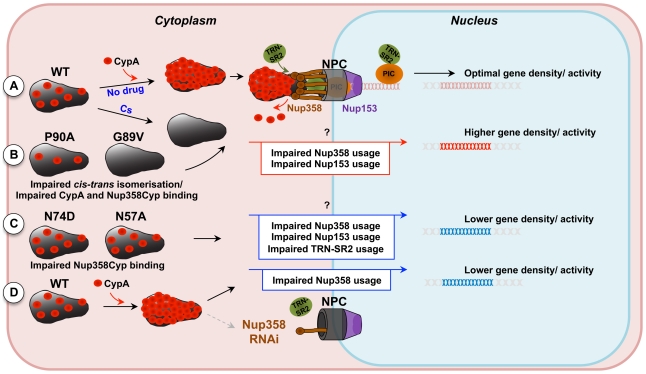
Model for CypA-dependent/-independent HIV-1 nuclear entry pathways. **A**, Wild type (WT) HIV-1 capsids are bound by cytoplasmic cyclophilin A (CypA) molecules that direct the capsid to use a specific nuclear entry pathway. The nuclear entry pathway involves CA binding to the cyclophilin domain of the cytoplasmic nuclear pore complex (NPC) component Nup358. Nup358 may mediate uncoating directly at the nuclear pore, liberating the pre-integration complex, which will interact with TRN-SR2 and the nuclear NPC component Nup153. **B**, Inhibition of CypA-CA interaction by the drug cyclosporine (Cs) or by substitution of CA residues that abolish CypA binding directs the virus into a less productive CypA-independent nuclear entry pathway that displays impaired dependence on Nup358 and Nup153. This route of nuclear entry results in virus integration preferences in areas of higher density of transcription units and associated features. **C**, HIV-1 capsid mutants that are less sensitive to Nup358, Nup153 or TRN-SR2 RNAi (HIV-1 CA N57A or N74D) enter the nucleus through a different pathway that directs their integration into genome areas of lower density of transcription units. **D**, Depletion of Nup358 by RNAi reduces viral nuclear entry via the Nup358-dependent pathway and the virus gains access via an Nup358-independent alternative pathway resulting in phenotypically similar integration site selection as observed for the CA mutants N74D or N57A. Alternative nuclear entry pathways disturb HIV-1 integration site selection, possibly contributing to sub-optimal replication of the virus in spreading infection assays.

Importantly, Cs treatment prevented spreading infection of wild type HIV-1 in human MDM [42](Figure 6D). Thus, productive infection in a biologically relevant cell type is dependent on the conserved use of cyclophilins. Our results suggest that inhibition of CypA may not only prevent the use of Nup358 but rather may direct HIV-1 into a Nup358/Nup153 independent nuclear entry pathway that may not be available or functional in MDM. In one possible model, for some cell lines such as HeLa cells, the use of alternate pathways and retargeting of integration preferences may not lead to large infectivity defects particularly when measuring infectivity using VSV-G pseudotyped HIV-1 vectors with GFP driven from a heterologous promoter. In replication assays using full length HIV-1 and primary targets of HIV-1 infection such as macrophages, Cs treatment (Figure 6D) or CA residue changes (Figure 4F) have their strongest inhibitory effects. Whilst our observations can be explained by various models, they support the notion that the cofactors that the virus has evolved to use, and has conserved the use of, such as Nup358 and TRN-SR2, may be most important in the primary cells in which the virus naturally replicates. A model is presented in cartoon form (Figure 7).

## Discussion

Here we have presented data suggesting that HIV-1 uses a pathway that includes the cytoplasmic cyclophilin CypA and the nuclear pore associated cyclophilin Nup358 to access the nucleus and target preferred regions of the genome for integration. We find that a determinant for the use of this pathway is CA and we demonstrate the first direct interaction of HIV-1 CA with a component of the nuclear pore complex. Disrupting engagement of CypA/Nup358 by mutating CA or inhibiting CypA with Cs appears to cause HIV-1 to use a Nup358/Nup153 independent pathway. The role of CypA in this process remains obscure but our data suggests that it directs HIV-1 to utilize a nuclear entry pathway involving Nup358 and Nup153. Indeed, roles for CypA in nuclear transport of cellular factors have been proposed before [43]–[45]. Our data illustrate that this HIV-1 nuclear import pathway is directly linked to integration site preference, which provides a candidate explanation for reduced replication in human MDM. Intriguingly, HIV-1 CA substitutions can influence the regions of the genome that the virus targets for integration. We have distinguished between the regions targeted for integration using criteria related to the density of transcription units, including GC content, DNAaseI hypersensitivity and gene expression. Infections with HIV-1 variants containing substitutions in CA that prevent CypA binding (e.g. G89V), and inhibiting CypA binding with Cs, both lead to increased frequency of integration in regions with higher densities of transcription units (Figure 4 and 5), supporting the consistency of our observations. Furthermore, mutations that render HIV-1 less sensitive to both Nup358 and TRN-SR2 depletion (CA N57A and N74D) both shift integration preferences to regions with lower densities of transcription units (Figure 4). Thus mutations that prevent HIV-1 utilizing Nup358 and TRN-SR2 have the same effect as depletion of these proteins, as described in our previous study [16]. These observations suggest that nuclear entry pathways may lead to different areas of chromatin and provide probes to investigate this possibility.

Several reports have suggested a role for HIV-1 CA in nuclear entry [7], [8], [10], [17], [40], [46]. Using ITC experiments we demonstrate here that HIV-1 binds to the nuclear pore through interactions between CA and the C-terminal Cyp domain of Nup358. This is the first direct evidence for an interaction between CA and the NPC and suggests that CA-containing PICs or whole capsid cores dock at the NPC prior to nuclear entry as previously inferred from microscopy studies [47]. Recruitment of cores through Nup358 may assist appropriate uncoating and interaction of PICs with the nuclear transport machinery including TRN-SR2 and Nup153. Remarkably, Nup358Cyp shows evidence for positive selection and a positively selected residue affects restriction by TRIMNup358 suggesting that this residue impacts on HIV-1 CA binding. This is the first case of an HIV-1 co-factor displaying signs of positive selection. We speculate that ancient pathogens, possibly viruses, may have provided the necessary selective pressure for the change of residue 61 from methionine, which has been conserved in the entire cyclophilin family, to valine, isoleucine or leucine in Nup358Cyp. It will be interesting to examine whether other viruses that encounter the nucleus during their life cycle use Nup358 and whether this position influences their recruitment. Indeed, Nup358 has been suggested to be involved in HSV-1 capsid attachment to the nucleus, however the viral determinants for this process remain obscure [48].

We also demonstrate that HIV-1 CA sequence influences the sites in which HIV-1 integrates. Although there are many possible explanations for that, we hypothesize that this occurs through selection of the cofactors for nuclear import or the nuclear import pathway. In the future, it will be interesting to investigate whether TRN-SR2 functions to enhance cytoplasmic availability of HIV-1 co-factors required for nuclear import or integration site selection. Interaction with such co-factors may be disturbed by CA mutations leading to impaired nuclear import or integration.

Surprisingly SIVmac, a primate lentivirus from rhesus macaques that was derived experimentally from SIV from sooty mangabeys [49] does not appear to utilize Nup358 during infection. SIVmac is however sensitive to TRN-SR2 depletion suggesting that it uses a related but somewhat different set of co-factors to enter the nucleus as compared to HIV-1. SIVmac is known to integrate into genes in a similar way to HIV-1 but subtle differences between HIV-1 and SIVmac integration targeting may exist. The significance of these observations remains unclear and characterization of the pathways used by a variety of lentiviruses to enter the nucleus and target favored sites will undoubtedly be informative.

Whilst our data don't rule out partial cytoplasmic uncoating we envisage the HIV-1 CA acting as a protective cage around the reverse transcription complex, shielding the viral macromolecules from pattern recognition by innate immune mediators present in the cytoplasm. Antagonistic Nup358 and CypA activities could be explained by a model in which CypA stabilizes or protects the core [50], whilst Nup358 regulates uncoating at the nuclear pore [47]. In this regard Nup358 binding to the conical viral core could have different effects from monomeric CypA, as eight Nup358 proteins are attached to the NPC [51]. Multiple simultaneous Nup358-CA interactions might destabilize the HIV-1 core and liberated PICs could then interact with TRN-SR2 and the nuclear located Nup153 ensuring transport through the NPC to appropriate sites [52]. This model provides a rationale for conservation of Cyp binding and explains how CA might influence TRN-SR2 or Nup153 usage without direct interaction [22], [40], [46], [52]. This model may also explain how the use of TRN-SR2 does not correlate with the ability of various integrase proteins to bind TRN-SR2 protein *in vitro* [46]. If lentiviruses regulate uncoating through interactions between CA and other host factors then their integrase proteins may be exposed to different karyopherins during this process. In this way the CA sequence and structure might be a stronger influence on the choice of karyopherins than integrase despite integrase being the ultimate target for karyopherin interaction. The Nup358 cyclophilin domain has been suggested to act as a chaperone by mediating prolyl *cis*-*trans* isomerization of cellular proteins [29], [30]. It will be interesting to investigate whether Nup358 is enzymatically active on the HIV-1 capsid core and whether this causes uncoating at the nuclear pore.

Currently available cyclosporins do not antagonize Nup358Cyp binding to HIV-1 CA but the fact that cyclophilins can be pharmacologically inhibited suggests the possibility of specifically inhibiting HIV-1 CA-Nup358Cyp interaction and possibly HIV-1 replication. Overall, our data demonstrate that rather than being lost during cytoplasmic uncoating, HIV-1 CA binds to the nuclear pore component Nup358 and directs the virus into a pathway that regulates its traffic between the cytoplasm and chromatin, playing a key role in the integration site targeting required for optimal continuation of the viral replication cycle.

## Materials and Methods

### Viruses, Vectors and Infection Assays

VSV-G pseudotyped vectors derived from HIV-1, SIVmac and MLV-B have been described as has their preparation by 293T transfection [53]. The HIV-1/SIVmac chimeric vectors have been described [20], [21] as has the HIV-1 vector encoding MVP5180 Gag [19]. HIV-1 NL4.3GFP-IRES has been described [54]. Viral doses were measured by reverse transcriptase (RT) enzyme linked immunosorbant assay (Roche). Viral vector infection assays using VSV-G pseudotyped viruses encoding GFP were analyzed by enumerating the number of green cells 48 hours post infection by flow cytometry. Viral vector infectivity experiments were performed in a 24-well plate format as described [53]. To measure late RT products, 2-LTR circles and integrated provirus, control or shRNA expressing cells were infected with VSV-G pseudotyped HIV-1 GFP encoding vector and then grown for indicated times. Total DNA was purified from 2 samples at each time point (QiaAmp, Qiagen) and 600 ng were subjected to Taqman quantitative PCR using late RT [55], 2-LTR circle [56] or GFP [53] primers and probe to detect provirus as described. Infectivity was measured in parallel samples by flow cytometry 48 hours post infection.

### Replication Assay in Human Monocyte Derived Macrophages (MDM) and TZM-bl Cells

MDM were prepared from fresh blood from healthy volunteers as described [57]. Cells were infected with 400 pg RT/well in 24-well plates and subsequently fixed and stained using a CA specific antibody (CA183) and a secondary antibody linked to beta galactosidase as described [57]. For measuring the effect of CypA inhibition on HIV-1 replication the assay was performed in the presence of 5 µM DMSO or Cs throughout the whole time course. TZM-bl infection assay was performed with 50 pg RT/20000 cells in 24-well plate dishes and RLU were measured at indicated time points.

### Integration Site Sequencing

Methods for integration site sequencing and heat map and dendogram analysis have been described [16].

### RNAi, Antibodies and Drugs

All RNA interference experiments were performed by expressing short hairpin RNA from either MLV vector pSIREN RetroQ (Clontech) (for Nup358, TRN-SR2 and Nup153) or pSUPER (Oligoengine) (for CypA) or if indicated from the HIV-1 vector pCSRQ, which was derived by subcloning the shRNA expression cassette from pSIREN RetroQ into pCSGW. The CypA shRNA target sequence has been described [58]. The Nup358 shRNA target sequence that was used throughout the study was 5-GCGAAGTGATGATATGTTT-3. Nup153 shRNA target sequence was 5-CAATTCGTCTCAAGCATTA-3. Both sequences were selected as 1 of the 4 target sequences from the Dharmacon siRNA smartpool for Nup358 or Nup153, respectively. Both shRNAs had only minor toxic effects on the cells, unlike shRNAs derived from the other three target sequences of each smart pool (Figure S1A, and data not shown). Additional Nup358 shRNA target sequences used in the experiment shown in Figure S1A were shRNA2 5-CAAACCACGTTATTACTAA-3, shRNA3 5-CAGAACAACTTGCTATTAG-3 and shRNA4 5-GAAGGAATGTTCATCAGGA-3. Specificity for each target sequence was confirmed by BLAT (UCSC genome browser). For Nup358, we confirmed effective targeting by co-transfecting the shRNA expression vector with a plasmid encoding GFP-tagged Nup358 (Figure S2), as well as by western blotting using a Nup358 specific antibody (Figure 1B). TRN-SR2 target sequence and control have been described and were also validated by co-transfecting a plasmid encoding for TRN-SR2-IRES-eGFP with the expression vectors encoding shRNA or control (SC) (Figure S2) [22]. The observations made for shRNA expressing HeLa cells were similar between populations of puromycin selected cells and clonal cells but the phenotype of cell clones was more stable, thus we used single cell clones for all experiments (Figure S1A, B and data not shown). Nup358, TRN-SR2, Nup153, CypA and beta-Actin were detected by western blot using a Nup358 antibody kindly given by Frauke Melchior, mouse TRN-SR2 antibody ab54353 (Abcam), mouse Nup153 antibody ab24700 (Abcam), rabbit CypA antibody SA296 (Biomol) and mouse beta-Actin antibody ab6276 (Abcam) and appropriate horseradish peroxidase linked secondary antibodies. TRIMCypA and TRIMNup358 were detected using anti-HA antibody 3F10 (Roche). Cyclosporine (Sandoz) and aphidicolin (Sigma) were diluted in DMSO and used at 5–8 µM and 2 µg/ml, respectively.

### Binding Assays and Positive Selection Analysis

Isothermal titration calorimetry was performed as described [25].

### Positive Selection Analysis

Codon-specific selection analysis was performed using the Random Effect Likelihood (REL) algorithm as described [27] using the alignment in Figure S6.

## Supporting Information

Figure S1 **Effects of Nup358 and transportin 3 RNAi on HIV-1 infectivity.** **A**, HeLa cells transiently transduced with the MLV vector pSIREN-RetroQ expressing four different shRNAs were infected in parallel with HIV-1 or MLV GFP encoding vector and infectious units per milliliter viral supernatant were determined. The target sequences for each shRNA were derived from the Dharmacon Smartpool. Three of the four shRNA vectors tested were cytotoxic and reduced MLV infectivity significantly. Vector encoding Nup358 shRNA1 was used for further experiments. **B**, NL4.3 derived VSV-G pseudotyped HIV-1 GFP vector was titrated on cell populations transiently transduced with lentiviral shRNA expression vectors CSRQ encoding scrambled control (SC), transportin 3 (TRN-SR2) or Nup358 specific shRNA and infectious units per ng RT were determined. **C**, VSV-G pseudotyped GFP encoding vectors derived from NL4.3 HIV-1, SIVmac, HIV-1 MVP5180 and MLV were titrated on representative HeLa cell clones expressing scrambled control (SC) or Nup358 or transportin 3 (TRN-SR2) specific shRNA. Graphs show the percentage of infected GFP positive cells per ng RT measured at 48 hours post infection. The data are representative of three independent experiments with three independent single cell clones.
(PDF)

Figure S2 **Validation of shRNA targeting efficiency.** **A**, Plasmid eGFPNup358 was co-transfected with vectors encoding shRNAs against scrambled control (SC), TRN-SR2 or Nup358 into 293T cells. Transfected cells were analyzed by flow cytometry and percentage of GFP-positive cells (left panels) as well as mean fluorescence intensity (MFI) of GFP-positive cells (right panels) was determined. **B**, Plasmid TRN-SR2-IRES-eGFP was co-transfected with indicated shRNA expression vectors into 293T and analysis was performed as in A.
(PDF)

Figure S3 **TRN-SR2 RNAi blocks HIV-1 at two steps.** HeLa cells stably transduced with vectors encoding shRNA targeting scrambled control (SC), TRN-SR2 or Nup358 were infected with HIV-1 GFP vector and grown for two weeks before extraction of total DNA. As a control cells were inoculated with boiled supernatant. Abundance of integrated provirus was measured from 500 ng total DNA of two independent samples per cell line by Taqman qPCR for GFP. Fold changes to infectivity on EV cells are shown above the bars. TRN-SR2 RNAi reduced 2-LTR circles by four-fold (Figure 1D), whereas reduction of integrated provirus was 50-fold, suggesting that there are two blocks in a canonical way, firstly at the step of nuclear entry and secondly at the step of integration. Boiled controls were below detectable levels.
(PDF)

Figure S4 **CD4 expression measured by antiCD4^APC^ staining.** HeLa control cells (SC), or cells expressing Nup358 or transportin 3 (TRN-SR2) specific shRNAs were transduced with MiGRI-CD4-IRES-neo MLV-vector and subsequently G-418 selected. CD4 expression in drug-selected populations was analyzed by staining with an antiCD4-antibody conjugated to APC as described [59]. As a negative control (NC) we used untransduced HeLa cells.
(PDF)

Figure S5 **HIV-1 CA mutants are impaired in Nup358Cyp binding.** CRFK cells expressing empty vector (EV) or TRIMNup358 were infected with the indicated GFP encoding VSV-G pseudotyped viral vectors and infectious titers per ng RT were determined. Fold changes to infectivity on EV cells are shown above the bars. The data are representative of two independent experiments each performed with three different vector doses (mean and SE, n = 2).
(PDF)

Figure S6 **Amino acid alignment of sequences used in the positive selection analysis.** Nup358Cyp DNA sequences from 12 different species [Rhesus macaque (*M. mulatta*), Human (*H. sapiens*), Chimpanzee (*P. troglodytes*), Dog (*C. familiaris*), Cow (*B. taurus*), Horse (*E. caballus*), Marmoset (*C. jacchus*), Mouse (*M. musculus*), Rat (*R. norvegicus*), Opossum (*M. domestica*), Orangutan (*P. abelii*) and Rabbit (*O. cuniculus*)] were aligned manually and translated into amino acid sequences. Analysis of codon-specific selective pressures using the algorithm Random Effect Likelihood (REL) implemented on the online version of the HyPhy package [28] was performed as previously described [27].
(PDF)

Figure S7 **Integration targeting of wild type HIV-1 and CA mutants.** **A**, We sequenced 19,546 unique integration sites by 454/Roche pyrosequencing and aligned them to the hg18 annotated human genome for analysis, as described [34]–[37]. We examined the density of several genomic features near integration sites, including relative gene density. Average of genes in a 1MB window around the integration sites were determined and compared to random controls for each virus. CypA/Nup358-independent CA mutants G89V, P90A and HIV-1(SIVCA) integrate in areas with higher gene density, whereas Nup358 and transportin 3-independent mutants N57A and N74D integrate in areas of gene density comparable to random controls. **B**, Wild type NL4.3 (Ba-L Env) or NL4.3 (Ba-L Env) bearing CA mutations P90A or N74D were used to infect human MDM from two donors in addition to those in Figure 4F at low multiplicity. Cells were stained for Gag p24 at specific time points after infection and infected colonies counted. Errors are standard error of the mean of three fields. Data are from independent virus preps on independent donors.
(PDF)

Figure S8 **Effect of CypA inhibition on Nup358 or transportin3 RNAi sensitivity.** HeLa cells expressing scrambled control (SC), Nup358 or transportin 3 (TRN-SR2) specific shRNA were infected with a fixed dose of wild type or mutant HIV-1 GFP in the presence of increasing concentrations of Cs (0–10 µM). Percentage infection was measured 48 hours later by detecting GFP positive cells using flow cytometry and plotted against Cs concentration.
(PDF)

Figure S9 **Effects of TRN-SR2 or Nup358 RNAi on HIV-1 Env pseudotyped vectors.** HeLa cells stably expressing scrambled control (SC), Nup358 or transportin 3 (TRN-SR2) specific shRNA were transduced with MLV vector MiGRI-CD4-IRES-neo and drug selected with G418. Similar CD4 expression was determined by antiCD4^APC^ staining (Figure S4). Generated cells were infected with HIV-1 GFP vectors that were pseudotyped with HIV-1 Env derived from YU-2. Infectivity of wild type HIV-1 (WT) was reduced similarly strongly as compared to VSV-G pseudotyped virus. HIV-1 CA mutants P90A and N74D showed a slightly increased sensitivity to both TRN-SR2 as well as Nup358 RNAi, suggesting envelope specific effects as suggested in a previous study for TRN-SR2 [31].
(PDF)

Table S1 **Effects of aphidicolin treatment.** Shown are fold reductions of infectious titers from graphs in Figure 3A. Left site of the table shows fold reduction of wild type HIV-1 or CA mutant infectious titer after aphidicolin (AC) treatment of HeLa cells transduced with vector encoding shRNA for scrambled control, Nup358 or TRN-SR2 as compared to untreated cells. Of note, infectious titers of all viruses, apart from N57A were not significantly changed in AC arrested cells as compared to untreated cells. Right site of the table shows fold reduction of infectious titers by Nup358 or TRN-SR2 RNAi as compared to control RNAi in AC arrested or untreated HeLa cells. Of note, the effect of Nup358 or TRN-SR2 RNAi on HIV-1 wild type virus (WT) or CA mutants, except N57A, was similar in AC arrested and untreated cells. N57A became slightly more sensitive to Nup358 or TRN-SR2 RNAi in arrested cells. However, N57A infectivity was reduced by ∼100 fold after AC treatment, suggesting that the 2–4 fold increased sensitivity to Nup358 or TRN-SR2 RNAi is not significant.
(PDF)
