# Supplementary figures and images for: HIV-1 Capsid-Cyclophilin Interactions Determine Nuclear Import Pathway, Integration Targeting and Replication Efficiency
Source: PLoS Pathog. 2011 Dec 8;7(12):e1002439. doi: 10.1371/journal.ppat.1002439 (PMC3234246; doi:10.1371/journal.ppat.1002439)

Figure S1

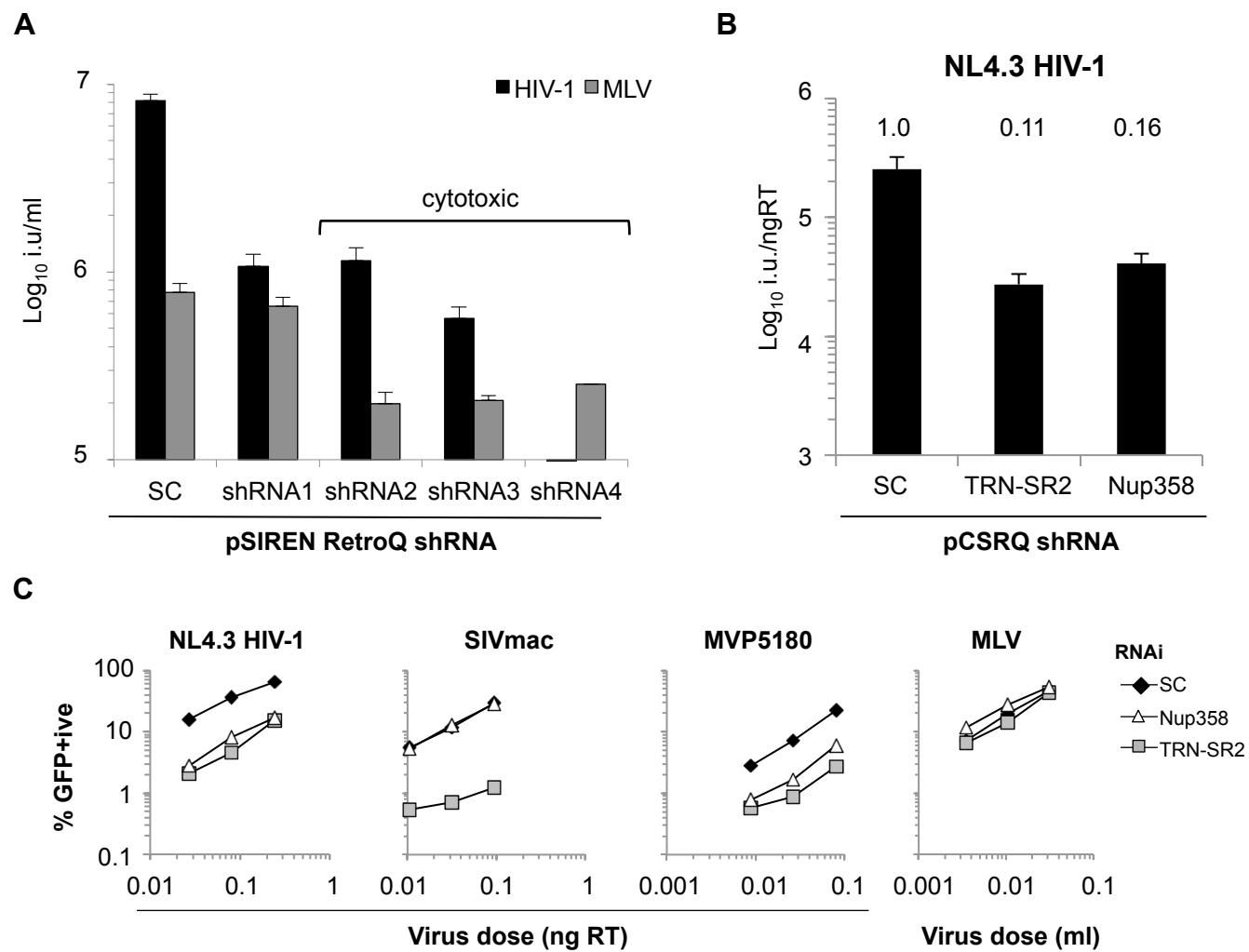

Supplement: Figure S1 — Effects of Nup358 and transportin 3 RNAi on HIV-1 infectivity. A, HeLa cells transiently transduced with the MLV vector pSIREN-RetroQ expressing four different shRNAs were infected in parallel with HIV-1 or MLV GFP encoding vector and infectious units per milliliter viral supernatant were determined. The target sequences for each shRNA were derived from the Dharmacon Smartpool. Three of the four shRNA vectors tested were cytotoxic and reduced MLV infectivity significantly. Vector encoding Nup358 shRNA1 was used for further experiments. B, NL4.3 derived VSV-G pseudotyped HIV-1 GFP vector was titrated on cell populations transiently transduced with lentiviral shRNA expression vectors CSRQ encoding scrambled control (SC), transportin 3 (TRN-SR2) or Nup358 specific shRNA and infectious units per ng RT were determined. C, VSV-G pseudotyped GFP encoding vectors derived from NL4.3 HIV-1, SIVmac, HIV-1 MVP5180 and MLV were titrated on representative HeLa cell clones expressing scrambled control (SC) or Nup358 or transportin 3 (TRN-SR2) specific shRNA. Graphs show the percentage of infected GFP positive cells per ng RT measured at 48 hours post infection. The data are representative of three independent experiments with three independent single cell clones. (PDF) [file ppat.1002439.s001.pdf]

Figure S2

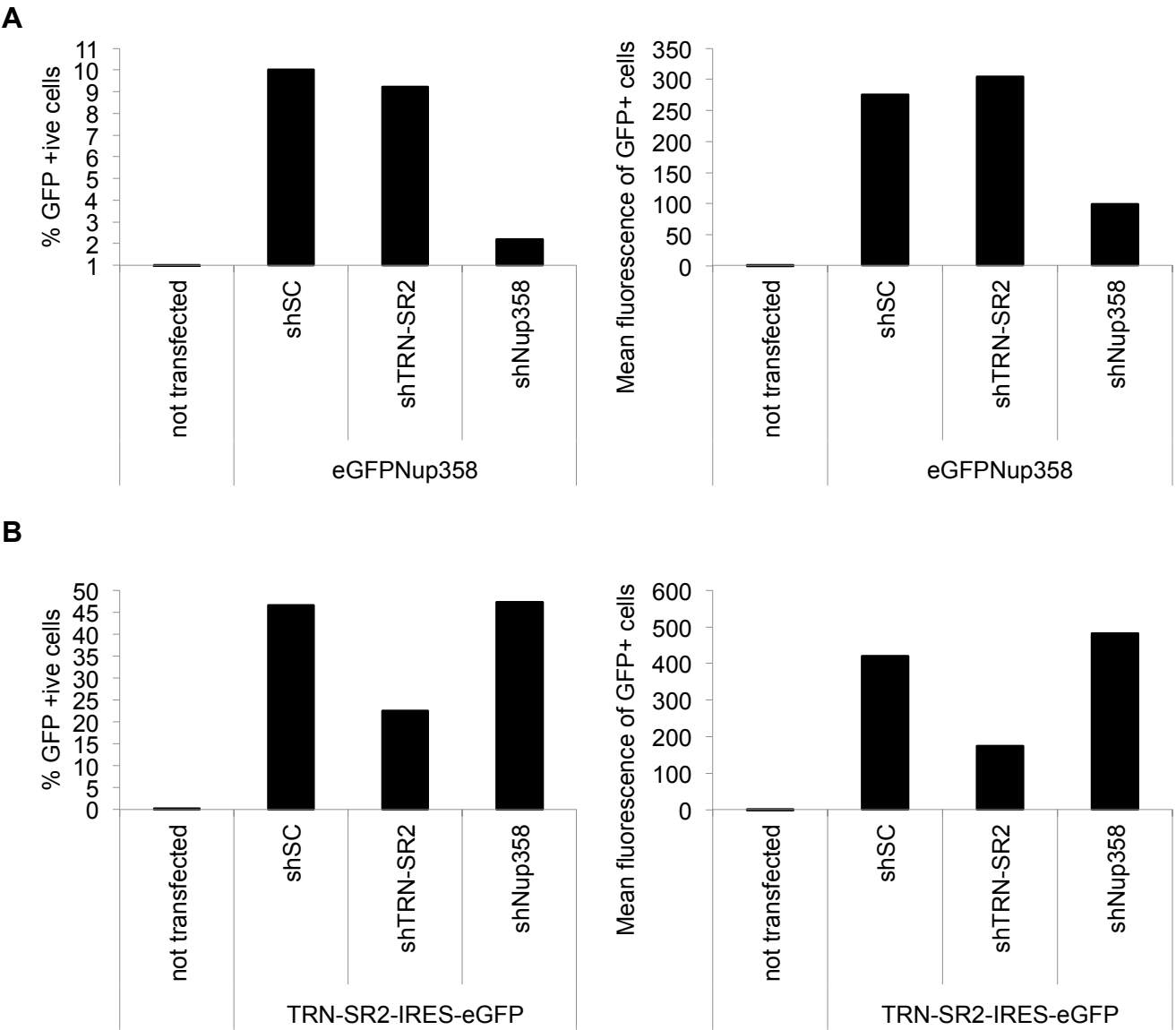

Supplement: Figure S2 — Validation of shRNA targeting efficiency. A, Plasmid eGFPNup358 was co-transfected with vectors encoding shRNAs against scrambled control (SC), TRN-SR2 or Nup358 into 293T cells. Transfected cells were analyzed by flow cytometry and percentage of GFP-positive cells (left panels) as well as mean fluorescence intensity (MFI) of GFP-positive cells (right panels) was determined. B, Plasmid TRN-SR2-IRES-eGFP was co-transfected with indicated shRNA expression vectors into 293T and analysis was performed as in A. (PDF) [file ppat.1002439.s002.pdf]

Figure S3

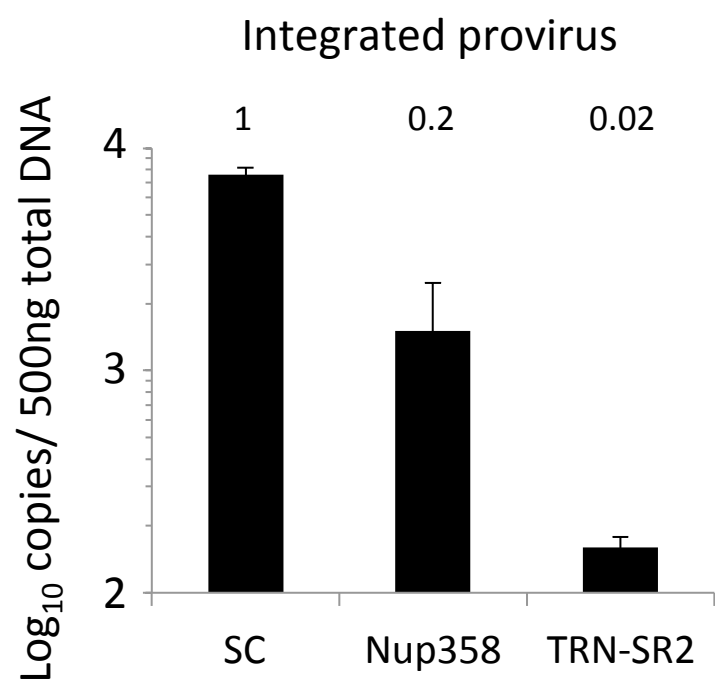

Supplement: Figure S3 — TRN-SR2 RNAi blocks HIV-1 at two steps. HeLa cells stably transduced with vectors encoding shRNA targeting scrambled control (SC), TRN-SR2 or Nup358 were infected with HIV-1 GFP vector and grown for two weeks before extraction of total DNA. As a control cells were inoculated with boiled supernatant. Abundance of integrated provirus was measured from 500 ng total DNA of two independent samples per cell line by Taqman qPCR for GFP. Fold changes to infectivity on EV cells are shown above the bars. TRN-SR2 RNAi reduced 2-LTR circles by four-fold (Figure 1D), whereas reduction of integrated provirus was 50-fold, suggesting that there are two blocks in a canonical way, firstly at the step of nuclear entry and secondly at the step of integration. Boiled controls were below detectable levels. (PDF) [file ppat.1002439.s003.pdf]

Figure S4

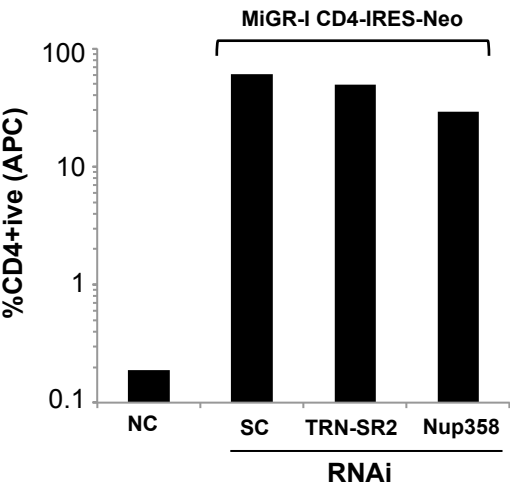

Supplement: Figure S4 — CD4 expression measured by antiCD4APC staining. HeLa control cells (SC), or cells expressing Nup358 or transportin 3 (TRN-SR2) specific shRNAs were transduced with MiGRI-CD4-IRES-neo MLV-vector and subsequently G-418 selected. CD4 expression in drug-selected populations was analyzed by staining with an antiCD4-antibody conjugated to APC as described [59]. As a negative control (NC) we used untransduced HeLa cells. (PDF) [file ppat.1002439.s004.pdf]

Figure S5

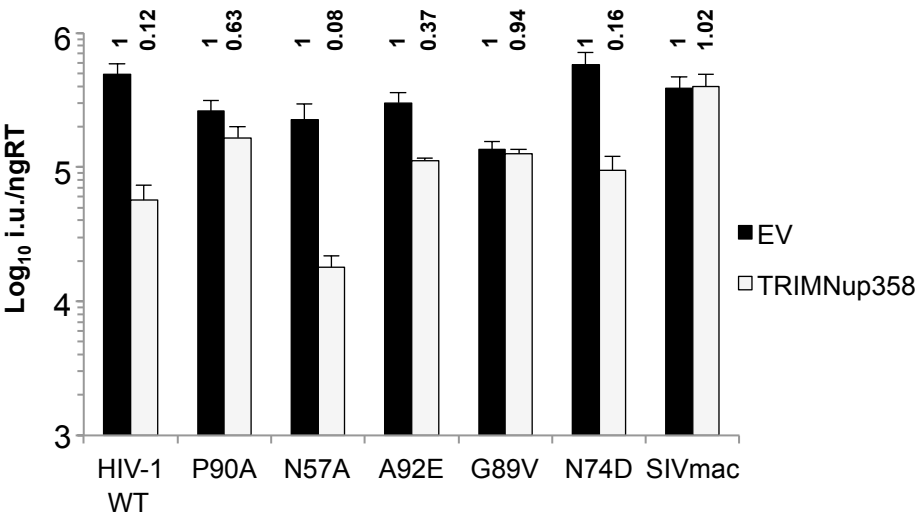

Supplement: Figure S5 — HIV-1 CA mutants are impaired in Nup358Cyp binding. CRFK cells expressing empty vector (EV) or TRIMNup358 were infected with the indicated GFP encoding VSV-G pseudotyped viral vectors and infectious titers per ng RT were determined. Fold changes to infectivity on EV cells are shown above the bars. The data are representative of two independent experiments each performed with three different vector doses (mean and SE, n = 2). (PDF) [file ppat.1002439.s005.pdf]

Figure S7

**A**

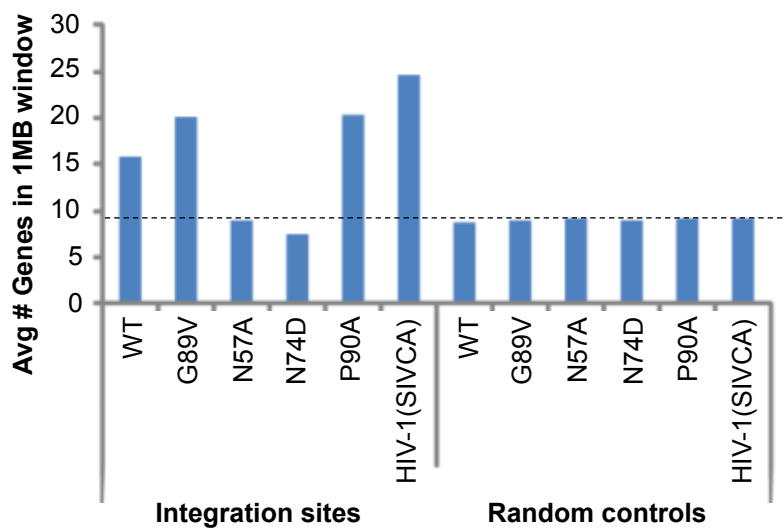

**B**

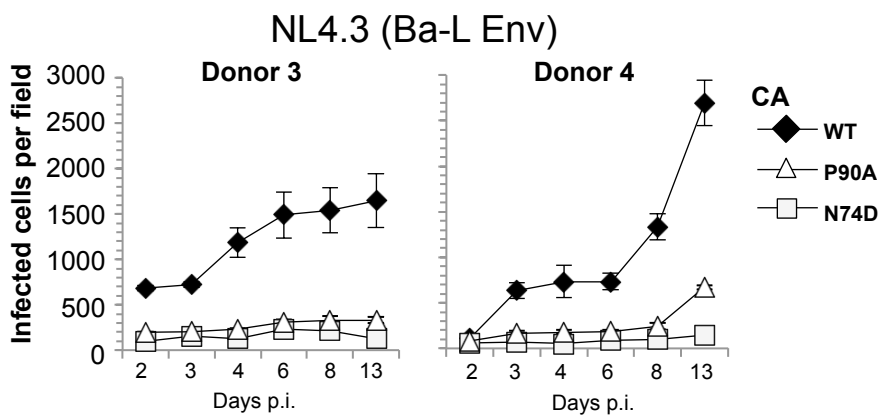

Supplement: Figure S7 — Integration targeting of wild type HIV-1 and CA mutants. A, We sequenced 19,546 unique integration sites by 454/Roche pyrosequencing and aligned them to the hg18 annotated human genome for analysis, as described [34]–[37]. We examined the density of several genomic features near integration sites, including relative gene density. Average of genes in a 1MB window around the integration sites were determined and compared to random controls for each virus. CypA/Nup358-independent CA mutants G89V, P90A and HIV-1(SIVCA) integrate in areas with higher gene density, whereas Nup358 and transportin 3-independent mutants N57A and N74D integrate in areas of gene density comparable to random controls. B, Wild type NL4.3 (Ba-L Env) or NL4.3 (Ba-L Env) bearing CA mutations P90A or N74D were used to infect human MDM from two donors in addition to those in Figure 4F at low multiplicity. Cells were stained for Gag p24 at specific time points after infection and infected colonies counted. Errors are standard error of the mean of three fields. Data are from independent virus preps on independent donors. (PDF) [file ppat.1002439.s007.pdf]

Figure S8

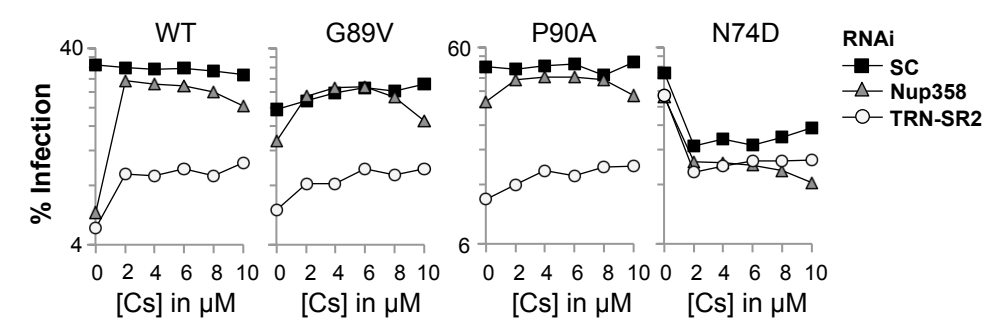

Supplement: Figure S8 — Effect of CypA inhibition on Nup358 or transportin3 RNAi sensitivity. HeLa cells expressing scrambled control (SC), Nup358 or transportin 3 (TRN-SR2) specific shRNA were infected with a fixed dose of wild type or mutant HIV-1 GFP in the presence of increasing concentrations of Cs (0–10 µM). Percentage infection was measured 48 hours later by detecting GFP positive cells using flow cytometry and plotted against Cs concentration. (PDF) [file ppat.1002439.s008.pdf]

Figure S9

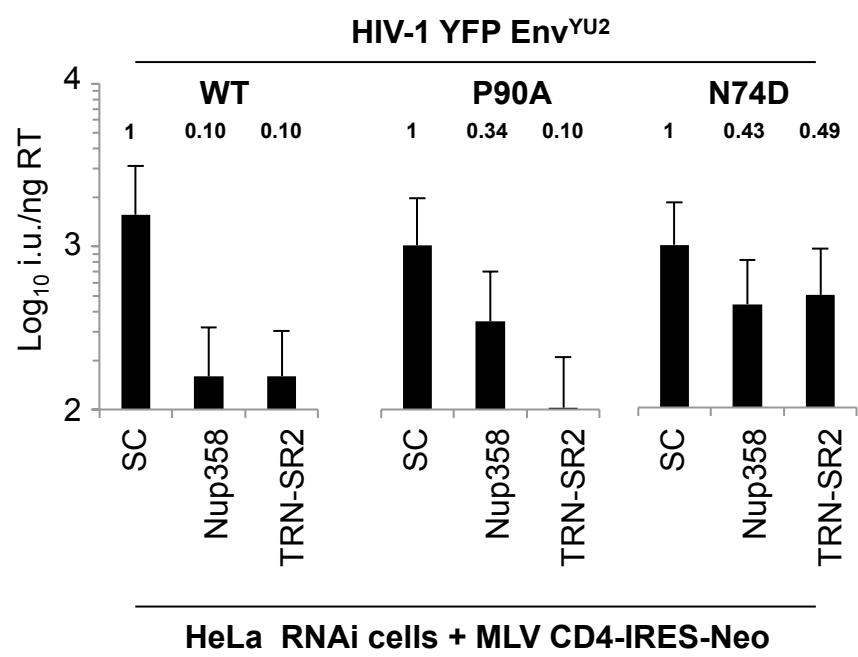

Supplement: Figure S9 — Effects of TRN-SR2 or Nup358 RNAi on HIV-1 Env pseudotyped vectors. HeLa cells stably expressing scrambled control (SC), Nup358 or transportin 3 (TRN-SR2) specific shRNA were transduced with MLV vector MiGRI-CD4-IRES-neo and drug selected with G418. Similar CD4 expression was determined by antiCD4APC staining (Figure S4). Generated cells were infected with HIV-1 GFP vectors that were pseudotyped with HIV-1 Env derived from YU-2. Infectivity of wild type HIV-1 (WT) was reduced similarly strongly as compared to VSV-G pseudotyped virus. HIV-1 CA mutants P90A and N74D showed a slightly increased sensitivity to both TRN-SR2 as well as Nup358 RNAi, suggesting envelope specific effects as suggested in a previous study for TRN-SR2 [31]. (PDF) [file ppat.1002439.s009.pdf]
