# Supplementary material for: HIV-1 Capsid-Cyclophilin Interactions Determine Nuclear Import Pathway, Integration Targeting and Replication Efficiency
Source: PLoS Pathog. 2011 Dec 8;7(12):e1002439. doi: 10.1371/journal.ppat.1002439 (PMC3234246; doi:10.1371/journal.ppat.1002439)
Supplement: Figure S6 — Amino acid alignment of sequences used in the positive selection analysis. Nup358Cyp DNA sequences from 12 different species [Rhesus macaque (M. mulatta), Human (H. sapiens), Chimpanzee (P. troglodytes), Dog (C. familiaris), Cow (B. taurus), Horse (E. caballus), Marmoset (C. jacchus), Mouse (M. musculus), Rat (R. norvegicus), Opossum (M. domestica), Orangutan (P. abelii) and Rabbit (O. cuniculus)] were aligned manually and translated into amino acid sequences. Analysis of codon-specific selective pressures using the algorithm Random Effect Likelihood (REL) implemented on the online version of the HyPhy package [28] was performed as previously described [27]. (PDF) [file ppat.1002439.s006.pdf]

Figure S6

```
                ....|....| ....|....| ....|....| ....|....| ....|....| ....|....|
                        10         20         30         40         50         60
M.mulatta       ETNPVVFFDV CADGEPLGRI TMELFSNIVP RTAENFRALC TGEKGFGFKN SIFHRVIPDF
H.sapiens       ETNPVVFFDV CADGEPLGRI TMELFSNIVP RTAENFRALC TGEKGFGFKN SIFHRVIPDF
P.troglodytes   ETNPVVFFDV CADGEPLGRI TMELFSNIVP RTAENFRALC TGEKGFGFKN SIFHRVIPDF
C.familiaris    ETNPVVFFDV CADDEPLGRI TMELFSNIVP LTAENFRALC TGEKGFGFKN SIFHRVIPDY
B.taurus        ETNPVVFFDI CADDEPLGRI TMELFSNIVP KTAENFRALC TGEKGFGFKS SIFHRVIPDF
E.caballus      ETNPVVFFDV CADDEPLGRI TMELFSNIVP RTAENFRALC TGEKGFGFKN SVFHRVIPGF
C.jacchus       ETNPVVFFDV CADSEPLGRI TMELFSNIVP RTAENFRALC TGEKGFGFKN SIFHRVIPDF
M.musculus      DTNPVVFFDV CADGEPLGRI IMELFSNIVP QTAENFRALC TGEKGFGFKN SIFHRVVPDF
M.domestica     ETNPVVFFEI FADDEHLGRI TIELFSNIVP LTAENFRALC TGERGFGFKN SVFHRVIPDF
P.abelii        ETNPVVFFDV CADGEPLGRI TMELFSNIVP RTAENFRALC TGEKGFGFKN SIFHRVIPDF
O.cuniculus     ETNPVVFFDI CADDEPLGRI TMELFSNIVP QTAENFRALC TGEKGFGFKN SIFHRVIPDC
R.norvegicus    ETNPVVFFDV CVDGEPLGRI IMELFSNIVP QTAENFRALC TGEKGFGFKN SIFHRVVPDF

                ....|....| ....|....| ....|....| ....|....| ....|....| ....|....|
                        70         80         90        100        110        120
M.mulatta       VCQGGDITKH DGTGGQSIYG DKFEDENFDV KHTGPGLLSM ANRGQNTNNS QFFITLKKAE
H.sapiens       VCQGGDITKH DGTGGQSIYG DKFEDENFDV KHTGPGLLSM ANQGQNTNNS QFVITLKKAE
P.troglodytes   VCQGGDITKH DGTGGQSIYG DKFEDENFDV KHTGPGLLSM ANQGQNTNNS QFFITLKKAE
C.familiaris    VCQGGDITKH DGTGGRSIYG DKFEDENFDV KHTGPGLLSM ANRGRDTNNS QFFITLKKAE
B.taurus        VCQGGDITKH DGTGGRSIYG DKFEDENFDV KHTGPGLLSM ANRGQDTNNS QFFITLKKAE
E.caballus      VCQGGDITKH DGTGGRSIYG DKFEDENFDV KHTDPGLLSM ANRGQDTNNS QFFIILKKAE
C.jacchus       VCQGGDITKH DGTGGQSIYG DKFEDENFDM KHTGPGLLSM ANRGQNTNNS QFFITLNKAE
M.musculus      ICQGGDITKY NGTGGQSIYG DKFDDENFDL KHTGPGLLSM ANYGQNTNSS QFFITLKKAE
M.domestica     ICQGGDITKH DGTGGRSIYG NTFEDENFTV RHTGPGLLSM ANRGRDTNNS QFFILLKKAE
P.abelii        VCQGGDITKH DGTGGQSIYG DKFEDENFDV KHGGPGLLSM ANQGQNTNNS QFFITLRKAE
O.cuniculus     LCQGGDITKH DGTGGKSIYG DKFEDENFDV KHTGPGLLSM ANRGPNTNNS QFFITLGKAE
R.norvegicus    ICQGGDITKY NGTGGQSIYG DKFDDENFDL KHTGPGLLSM ANCGQNTNSS QFFITLKKAE

                ....|....| ....|....| ....|....| ....|....| ....
                       130        140        150        160
M.mulatta       HLDFKHVVFG FVKDGMDTVK KIESFGSPKG SVCRRITITE CGQI
H.sapiens       HLDFKHVVFG FVKDGMDTVK KIESFGSPKG SVCRRITITE CGQI
P.troglodytes   LLDFKHVVFG FVKDGMDTVK KIESFGSPKG SVCRRITITE CGQI
C.familiaris    HLDFKHVVFG FVKDGMDTVK KIESFGSPKG SVSRRISITE CGQI
B.taurus        RLDFKHVVFG FVKDGMDTVK KIESFGSPKG SVSRRIIITE CGQI
E.caballus      HLDLKHVVFG FVKDGMDTVK KIESFGSLEG SVSRRISITE CGQI
C.jacchus       HLDFKHVVFG YVKDGMDTVK KIESFGSPKG SVGRRIAITE CGQI
M.musculus      HLDFKHVVFG FVKDGMDTVR KIESFGSPKG SVSRRICITE CGQL
M.domestica     HLDFKHVVFG FVKDGMDTVK KIESFGSPKG LVSKRIMITE CGQI
P.abelii        HLDFKHVVFG FVKDGMDTVK KIESFGSPKG SVCRRITITE CGQI
O.cuniculus     HLDFKHVVFG FVKDGMDTVK KIESFGSPKG SVSRRISITE CGQI
R.norvegicus    HLDFKHVVFG FVKDGMDTVR KIESFGSPKG SVSRRICITE CGQL
```
