# Supplementary material for: HIV-1 Capsid-Cyclophilin Interactions Determine Nuclear Import Pathway, Integration Targeting and Replication Efficiency
Source: PLoS Pathog. 2011 Dec 8;7(12):e1002439. doi: 10.1371/journal.ppat.1002439 (PMC3234246; doi:10.1371/journal.ppat.1002439)
Supplement: Table S1 — Effects of aphidicolin treatment. Shown are fold reductions of infectious titers from graphs in Figure 3A. Left site of the table shows fold reduction of wild type HIV-1 or CA mutant infectious titer after aphidicolin (AC) treatment of HeLa cells transduced with vector encoding shRNA for scrambled control, Nup358 or TRN-SR2 as compared to untreated cells. Of note, infectious titers of all viruses, apart from N57A were not significantly changed in AC arrested cells as compared to untreated cells. Right site of the table shows fold reduction of infectious titers by Nup358 or TRN-SR2 RNAi as compared to control RNAi in AC arrested or untreated HeLa cells. Of note, the effect of Nup358 or TRN-SR2 RNAi on HIV-1 wild type virus (WT) or CA mutants, except N57A, was similar in AC arrested and untreated cells. N57A became slightly more sensitive to Nup358 or TRN-SR2 RNAi in arrested cells. However, N57A infectivity was reduced by ∼100 fold after AC treatment, suggesting that the 2–4 fold increased sensitivity to Nup358 or TRN-SR2 RNAi is not significant. (PDF) [file ppat.1002439.s010.pdf]

Table S1

HIV-1	Fold reduction by AC treatment			Fold reduction by Nup358 RNAi		Fold reduction by TRN-SR2 RNAi	
	Control RNAi	*Nup358 RNAi*	*TRN-SR2 RNAi*	*No drug*	*AC*	*No drug*	*AC*
**WT**	2.12	2.35	1.00	5.70	6.31	7.70	3.65
**G89V**	1.22	0.98	1.16	1.52	1.23	4.05	3.84
**P90A**	1.29	1.02	0.88	1.38	1.09	4.62	3.15
**N74D**	0.94	0.97	1.37	1.18	1.22	1.13	1.64
**N57A**	31.64	129.09	73.48	0.94	3.82	0.99	2.29
